# Investigation of MMP-3, IL-6, IL-6R, and TNF-Alpha Levels in Fibromyalgia Patients Presenting to a Physical Medicine and Rehabilitation Clinic

**DOI:** 10.3390/jcm15114107

**Published:** 2026-05-26

**Authors:** Abdullah Gumus, Faruk Erdogan, Sermin Durak, Murat Karamese, Umit Zeybek

**Affiliations:** 1 Department of Medical Genetics, Faculty of Medicine, Kafkas University, Kars 36100, Türkiye; 2 Physical Medicine and Rehabilitation Clinic, Erzurum City Hospital, Erzurum 25240, Türkiye; frkerd25@gmail.com; 3 Department of Medical Microbiology, Faculty of Medicine, Istanbul University-Cerrahpaşa, Istanbul 34098, Türkiye; 4 Department of Medical Microbiology, Faculty of Medicine, Kafkas University, Kars 36100, Türkiye; 5 Department of Molecular Medicine, Aziz Sancar Experimental Medicine Research Institute, Istanbul University, Istanbul 34093, Türkiye

**Keywords:** interleukin-6, interleukin-6 receptor, TNF-α, matrix metalloproteinase-3, fibromyalgia

## Abstract

**Background:** Fibromyalgia syndrome (FMS) is a syndrome that commonly affects the musculoskeletal system and is characterized by fatigue and body aches. Recent studies have demonstrated a relationship between FMS and certain immunological markers. In this study, we investigated the relationship between MMP-3, IL-6, IL-6R, and TNF-α serum levels and disease impact (FIQ score) in FMS patients. **Methods:** This case–control study consisted of 164 participants, of whom 82 were patients with fibromyalgia syndrome (FMS) and 82 were healthy controls without FMS, chronic pain, or inflammatory diseases. Disease impact was assessed using the Fibromyalgia Impact Questionnaire (FIQ). MMP-3, IL-6, IL-6R, and TNF-α serum levels were detected using enzyme-linked immunosorbent assay (ELISA) kits. **Results:** In patients diagnosed with FMS, TNF-α, IL-6, IL-6R, and MMP-3 levels were significantly higher than those of the control group (all *p* < 0.0001). MMP-3, IL-6, IL-6R, and TNF-α levels correlated positively with the FIQ score. **Conclusions:** This study highlights the involvement of inflammatory processes in FMS, particularly the role of serum IL-6, TNF-α, IL-6R, and MMP-3 levels in the pathophysiology of FMS. The results indicate that inflammatory markers may be associated with the severity of FMS and may have potential as disease markers.

## 1. Introduction

Fibromyalgia syndrome (FMS) is a complex syndrome characterized by widespread body pain and fatigue [1,2]. It is typically associated with generalized musculoskeletal pain, numbness, sleep disturbances, anxiety, and/or depression, the severity and scale of which may vary throughout the course of the disease. FMS does not pose a life-threatening risk [3]. Physical sequelae are generally absent, but this disease can seriously affect and impair quality of life. Although many causes are thought to be involved, including infection, physical trauma, stress, emotional shock, and genetics, its etiology is not fully understood [4,5].

Epidemiological studies on FMS report a prevalence of 5% in women and 1.6% in men, commonly occurring in individuals aged 40–60 years. Although its etiopathogenesis has not yet been fully explained, various factors have been suggested to play a role, including sleep disturbance, neuroendocrine and hormonal dysfunction, regional blood flow changes, and metabolic and immunological disorders [3]. Historically, the 1990 ACR criteria relied on widespread pain lasting more than 3 months together with tenderness at 11 of 18 tender points [6], whereas the revised 2016 criteria no longer require tender-point examination [7]. Fibromyalgia is often accompanied by other symptoms, such as psychiatric disorders, headache, sleep disturbance, morning stiffness, paranesthesia, gastrointestinal dysfunction, chest pain, orthostatic hypotension, and tachycardia [8,9].

The factors involved in the pathophysiology of FMS have not been fully characterized, but studies have shown that immunological markers may yield effective results in diagnosis [10,11]. Cytokines play an important role in the development and differentiation of T cells, B cells, and hematopoietic cells, as well as in the induction or suppression of inflammation [11,12]. Some cytokines have local effects, while others have more pronounced systemic effects. Due to their impact on both the innate and adaptive immune systems and their systemic effects, they play a significant role in the pathogenesis of inflammatory diseases, making them attractive therapeutic targets [13,14,15]. Recent studies have shown that cytokines play an important role in the pathogenesis of FMS, particularly with an increase in pro-inflammatory cytokines [16,17]. In this study, we aim to assess the relationships between IL-6, TNF-α, IL-6R, and MMP-3 serum levels and disease severity (FIQ) in patients newly diagnosed with FMS and healthy controls.

## 2. Materials and Methods

### 2.1. Study Design and Participants

This case–control study comprised 164 subjects, of whom 82 were patients with fibromyalgia syndrome and 82 were healthy controls. The study was approved by the Non-Interventional Ethics Committee of Kars Kafkas University Faculty of Medicine (No: 80576354-050-99/272) and conducted in accordance with the Declaration of Helsinki, the Good Clinical Practice Guidelines, and relevant legislation. Patients were included if they had fibromyalgia syndrome, as defined by the 2016 American College of Rheumatology criteria [8], and attended the Physical Medicine and Rehabilitation Clinic of Kars Harakani State Hospital, Kars, Türkiye. Venous blood samples were collected from all subjects during their initial clinical examination and represent the earliest measurement taken at the moment of diagnosis. The blood sample was taken as part of a routine clinical procedure without any standardization regarding the time of collection within a day. Subsequently, samples were spun to obtain serum, which was then stored at −80 °C before the start of the experiment. All indicators were measured using commercially available ELISA kits (MyBioSource, San Diego, CA, USA). The exclusion criteria included systemic infections, rheumatological disease, malignancy, known chronic inflammatory or metabolic diseases that could affect inflammatory biomarker levels, physician-assessed obesity identified during routine clinical evaluation, steroid use, pregnancy, breastfeeding, and cognitive impairment. Controls were healthy subjects without fibromyalgia syndrome or a history of chronic pain or rheumatological disease within the last six months and were matched to the patients in terms of age and sex.

### 2.2. ELISA Analysis of Serum Biomarkers

Venous blood samples were collected from all participants. Routine laboratory tests were performed, and serum samples were collected via centrifugation and stored at −80 °C until analysis. Serum levels of IL-6, TNF-α, IL-6R, and MMP-3 were detected using commercially available ELISA kits (MyBioSource, San Diego, CA, USA).

### 2.3. Statistical Analysis

Statistical analysis was carried out using IBM SPSS Statistics, version 31.0 (IBM Corp., Armonk, NY, USA). The data are expressed as mean and standard deviation. Normality of data distribution was checked using the Shapiro–Wilk normality test. Since most of the data were not normally distributed, non-parametric tests were used for analysis. The differences between two independent groups were analyzed using the Mann–Whitney U test, whereas correlations were analyzed using Spearman’s correlation test. The diagnostic potential of biomarkers was checked via receiver operating characteristic curve analysis (ROC). Multiple regression analysis was used to check the independent effects of variables on FIQ score. *p*-values < 0.05 were considered significant.

## 3. Results

A total of 164 participants were enrolled in the study, including 82 patients with FMS and 82 healthy controls. The FMS group consisted of 62 females (75.6%) and 20 males (24.4%), whereas the control group comprised 60 females (73.2%) and 22 males (26.8%). The mean age was 48.44 ± 8.82 years in the FMS group and 47.46 ± 8.66 years in the control group. No statistically significant differences were observed between the groups in terms of age or gender distribution (*p* > 0.05). Although the FIQ score was normally distributed in the patient group, most biochemical markers (TNF-α, IL-6, IL-6R, MMP-3) were not normally distributed; therefore, non-parametric tests (Mann–Whitney U and Spearman correlation) were used at this step. In the FMS group, the mean FIQ score was 58.3 ± 13.7, while it was 20.1 ± 11.0 in the control group. The FIQ score was higher in patients with FMS than in the control group (*p* < 0.001) (Table 1).

**Figure 1:**
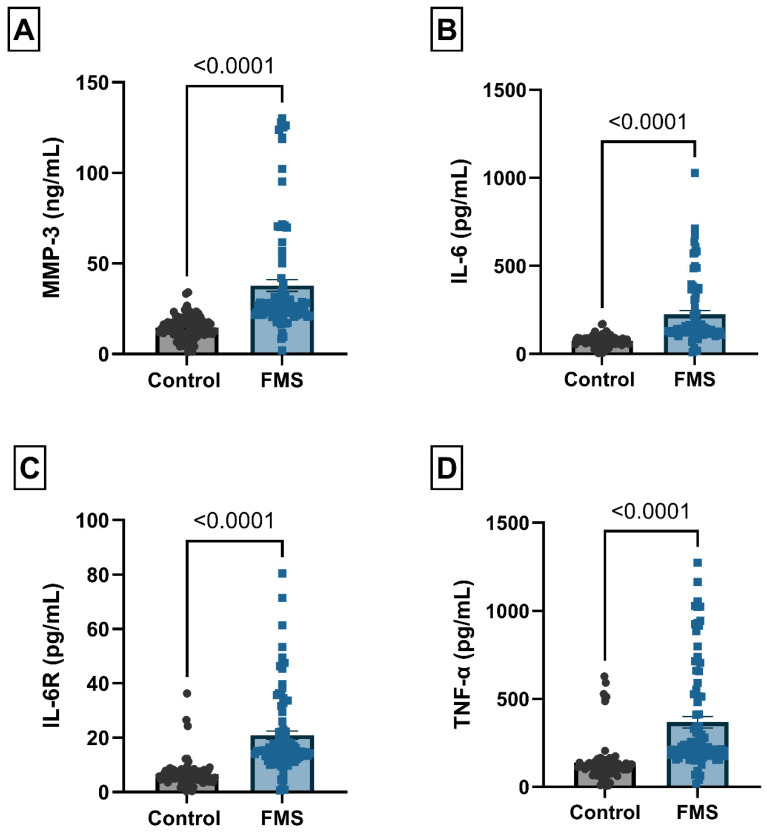
Comparison of inflammatory biomarker serum levels in FMS and control groups. (**A**) MMP-3 levels, (**B**) IL-6 levels, (**C**) IL-6R levels, and (**D**) TNF-α levels in the fibromyalgia syndrome patient group are significantly higher than those in the healthy control group. The results are depicted as individual values with bars. The Mann–Whitney U test was used to compare the groups. *p* < 0.0001.

As shown in Figure 1, TNF-α serum levels were significantly higher in patients with FMS than in the control group (367.6 ± 297.9 vs. 136.8 ± 116.4, *p* < 0.0001). MMP-3 levels (52.7 ± 39.3 vs. 15.3 ± 6.7, *p* < 0.0001), IL-6 levels (226.3 ± 183.7 vs. 74.6 ± 30.3, *p* < 0.0001), and IL-6R levels (20.9 ± 14.8 vs. 6.7 ± 4.9, *p* < 0.0001) were also significantly higher in the patient group than in the control group (Table 1).

**Figure 2:**
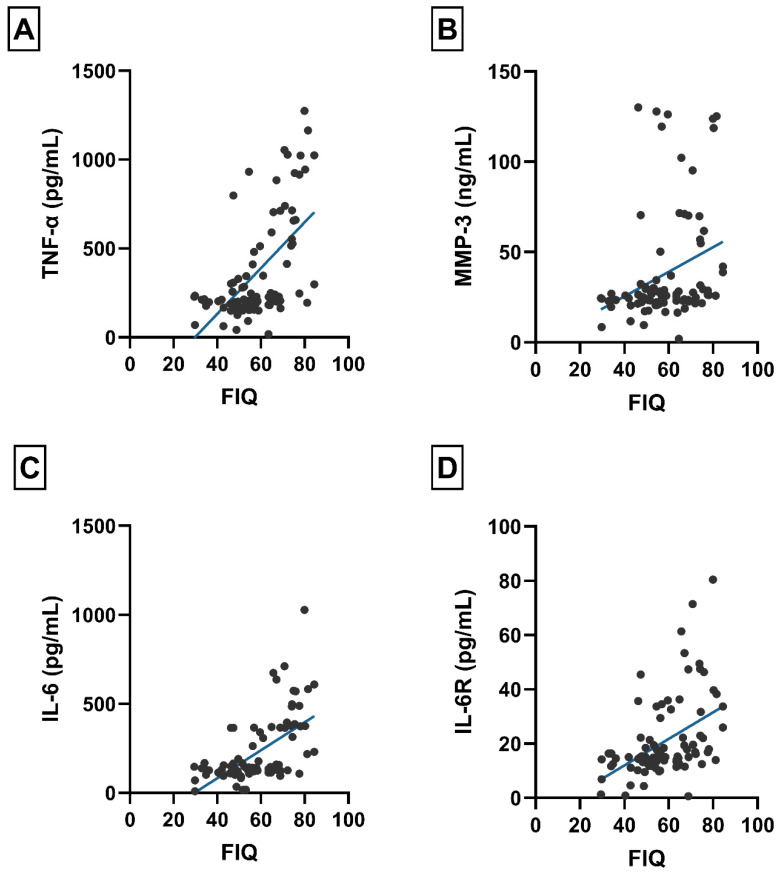
Correlation between FIQ score and serum inflammatory biomarkers in patients with fibromyalgia syndrome. The correlation between Fibromyalgia Impact Questionnaire (FIQ) score and serum concentrations of (**A**) TNF-α, (**B**) MMP-3, (**C**) IL-6, and (**D**) IL-6R in the patient group is demonstrated by scatter plots. Positive correlations were found between the FIQ score and all measured inflammatory biomarkers. Increased concentrations of the measured inflammatory biomarkers correlate with increased disease impact. Correlation analysis was performed using Spearman’s rank correlation test.

In the correlation analysis carried out within the patient group, the FIQ score showed moderate positive correlations with TNF-α (r = 0.540; *p* < 0.0001), IL-6 (r = 0.551; *p* < 0.0001), and IL-6R (r = 0.497; *p* < 0.0001), and a weak positive correlation with MMP-3 (r = 0.368; *p* = 0.0004) (Table 2). Moreover, statistically significant positive correlations between the FIQ score and all biomarkers were found in women (*p* < 0.001), while a significant correlation was found between IL-6 and the FIQ score in men (r = 0.526; *p* = 0.017). As shown in Figure 2, among the routine biochemical parameters, CK showed a strong positive correlation with the FIQ score (r = 0.654, *p* < 0.001), while vitamin D showed a strong negative correlation (r = −0.620, *p* < 0.001). Uric acid showed a moderate positive correlation with the FIQ score (r = 0.467, *p* < 0.001), while vitamin B12 showed a weak negative correlation (r = −0.215, *p* = 0.043). AST, ALT, fT4, and TSH did not show any significant correlations with the FIQ score (*p* > 0.05) (Table 3).

**Figure 3:**
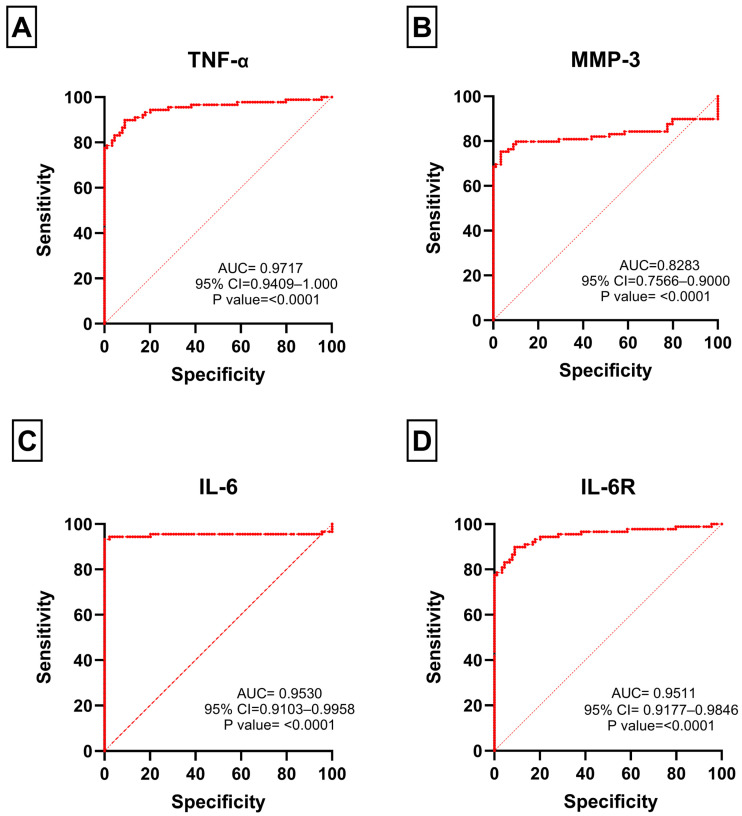
Receiver operating characteristic (ROC) curve analysis of inflammatory biomarkers for differentiating fibromyalgia syndrome patients and healthy controls. ROC curve analysis was performed for (**A**) TNF-α, (**B**) MMP-3, (**C**) IL-6, and (**D**) IL-6R. The area under the curve (AUC), 95% confidence interval (CI), and *p*-values were calculated for the ROC curve analysis. IL-6, IL-6R, and TNF-α show excellent diagnostic potential, while MMP-3 shows good diagnostic potential.

Multiple linear regression analysis indicated that IL-6 was the strongest predictor of FIQ score (*p* < 0.01), followed by TNF-α, MMP-3, and IL-6R also showing significant positive associations with disease severity (*p* < 0.05). The regression analysis accounted for 40% of the variance in FIQ score with an R^2^ value of 0.403. Receiver operating characteristic curve analysis was carried out to evaluate the diagnostic potential of TNF-α, MMP-3, IL-6, and IL-6R in differentiating patients with FMS from healthy controls. The results revealed that all markers under investigation showed high diagnostic accuracy. As shown in Table 4, the results showed that the diagnostic potential of TNF-α was 0.9717, that of MMP-3 was 0.8283, that of IL-6 was 0.9530 and that of IL-6R was 0.9511. According to standard ROC interpretation criteria, AUC values above 0.90 indicate excellent diagnostic performance, whereas values between 0.80 and 0.90 indicate good diagnostic performance. TNF-α, IL-6, and IL-6R demonstrated excellent diagnostic potential, whereas MMP-3 demonstrated good diagnostic potential (Figure 3).

## 4. Discussion

In the present study, we performed a detailed investigation of the correlation between systemic inflammatory markers (IL-6, TNF-α, IL-6R, and MMP-3) and disease severity as defined by the FIQ in patients with FMS. Our findings show that all of these biomarkers were statistically significantly higher in FMS patients than in healthy controls. Moreover, correlation and regression analyses showed that these biomarkers were positively correlated with disease severity; the ROC analysis showed good diagnostic potential of these biomarkers, especially for IL-6R and IL-6. These findings support the hypothesis of low-grade systemic inflammation in the pathogenesis of FMS.

Our results concur with recent meta-analyses indicating increased circulating levels of pro-inflammatory cytokines in FMS patients, especially IL-6 and TNF-α [18,19]. IL-6 plays a critical role in communication between the nervous system and immune system, particularly in central sensitization, fatigue, and pain, all of which are major components of FMS [20,21]. The significant correlation we found between IL-6 circulating levels and FIQ score further reinforces IL-6 as a major contribution to the pathophysiology of this disease. Likewise, TNF-α has been implicated in the sensitization of nociceptive pathways through both peripheral and central sensitization, particularly in the development of chronic pain [22,23]. The moderate to high correlation values we calculated for this cytokine also indicate its relevance in the overall burden of this disease.

The most striking observation of the present study is the high diagnostic potential of IL-6R, which had the highest AUC value of all biomarkers. This observation is particularly interesting since IL-6 signaling is not only mediated by the classical signaling pathway but also provides evidence of trans-signaling from soluble IL-6R. In fact, the evidence suggests that IL-6 trans-signaling is particularly crucial in the context of chronic inflammatory and pain conditions [24]. This is particularly interesting since IL-6R levels might indicate a more comprehensive activation of the IL-6 signaling pathway compared with IL-6. The high diagnostic accuracy observed for IL-6R and IL-6 suggests that these biomarkers may have utility as part of a future diagnostic panel for fibromyalgia syndrome. This approach could provide supplementary objective information to clinicians, particularly in cases with complex clinical presentations.

In addition to cytokines, the current study also showed a marked increase in MMP-3 levels in patients with FMS. Although matrix metalloproteinases have been extensively studied in inflammatory and degenerative diseases, their role in fibromyalgia is poorly understood. Recent studies indicate that MMP-3 could play a role in the mechanisms of extracellular matrix remodeling and peripheral sensitization [19,25,26]. These results provide evidence of tissue remodeling’s role in the pathophysiology of FMS and highlight the potential of MMP-3 as a new biomarker for this disease.

Significantly, multiple linear regression analysis revealed that the strongest predictor of FIQ score was IL-6, but TNF-α, MMP-3, and IL-6R also had significant effects. The model accounted for approximately 40% of the variance in disease severity. This suggests that inflammatory processes may play a significant but by no means exclusive role in the pathogenesis of FMS. Although the regression analysis explained approximately 40% of the variance in FIQ scores, a substantial proportion of the variability remained unexplained. This finding further supports the multifactorial nature of FMS, suggesting that non-inflammatory mechanisms, such as neuroendocrine dysregulation, psychological factors, sleep disturbances, central sensitization, and lifestyle-related variables, may also contribute significantly to disease burden and symptom severity. Therefore, our findings suggest that although systemic inflammation is an important contributor to FMS, it likely represents only one component of the complex pathophysiology underlying the disease [3,22,25].

The stratified analysis by gender showed that all biomarkers were correlated with disease severity in female patients, whereas only IL-6 showed a significant correlation in male patients. However, these findings should be interpreted cautiously because of the relatively small number of male participants, which may have limited the statistical power of the subgroup analyses. Therefore, these gender-specific findings should be considered exploratory and require confirmation in larger and more gender-balanced cohorts. A recent study has pointed out sex differences related to immune response and pain, which may explain some of these findings, as mentioned by Meester et al. (2020) [27].

Apart from inflammatory markers, significant correlations were also found between FIQ score and routine biochemical tests. CK levels demonstrated a strong positive correlation with FIQ score, so it is possible that muscle metabolism is related to the pathogenesis of this disease. Furthermore, a strong negative correlation was found between vitamin D levels and FIQ score. Recent studies have emphasized the effects of vitamin D deficiency on chronic pain and musculoskeletal disorders [28,29]. Furthermore, our findings highlight the potential importance of metabolic and nutritional factors in the management of fibromyalgia syndrome. In particular, the observed inverse relationship between vitamin D levels and FIQ score suggests a possible association between vitamin status and disease severity. However, further research is needed to clarify the clinical significance of these associations. Consistent with the literature, uric acid demonstrated a moderate positive correlation, while vitamin B12 revealed a weak negative correlation with FIQ score [30]. Thus, metabolic and nutritional factors also play a role in the pathogenesis of this disease.

In our study, CRP was not found to be correlated with FIQ scores. This observation is consistent with previous studies suggesting that CRP, a classical acute-phase reactant, may not consistently reflect symptom severity in FMS patients [11,31]. Moreover, previous study and meta-analyses have reported elevated CRP levels in patients with FMS compared with healthy controls, and CRP does not appear to consistently reflect disease-specific clinical manifestations or severity [32]. Although IL-6 and TNF-α have been implicated in the pathogenesis of FMS through mechanisms related to neuroinflammation and central sensitization, CRP represents a more nonspecific downstream marker of systemic inflammation [17,33]. Therefore, the lack of association observed in our study may suggest that CRP has limited utility in reflecting disease severity in FMS compared with cytokine-related biomarkers [20].

One of the major advantages of this study is that it uses a variety of analytical tools, such as non-parametric group comparison tests, correlation tests, regression analysis, and ROC curve analysis, providing a comprehensive evaluation of the diagnostic and prognostic potential of this disease. To the best of our knowledge, this is the first study evaluating the inflammatory and biochemical tests of patients with FMS from the northeastern Anatolia (Kars) region of Turkey. However, several limitations should be acknowledged. First, the relatively small and geographically limited sample may restrict the generalizability of the findings and introduce selection bias. Second, the relatively small number of male participants may have limited the statistical power of the gender-stratified subgroup analyses; therefore, these findings should be considered exploratory. Furthermore, blood sample collection was not standardized with respect to the time of day, which may have introduced pre-analytical variability, particularly for IL-6, a cytokine known to exhibit diurnal variation. In addition, although major confounding conditions such as known chronic inflammatory or metabolic diseases and physician-assessed obesity identified during routine clinical evaluation were excluded at enrollment, exact BMI values, disease duration, the broader comorbidity burden, and current medication use were not systematically recorded and may have influenced the interpretation of inflammatory biomarker levels. Finally, the cross-sectional design precludes causal inference, and the findings have not been externally validated in independent cohorts. Therefore, larger, multicenter longitudinal studies incorporating broader clinical phenotyping and multi-omics approaches are needed to better define the pathophysiology and clinical utility of these biomarkers in FMS.

In conclusion, the results of the current study clearly show a strong association between inflammatory biomarkers, especially IL-6 and IL-6R, and the presence and severity of fibromyalgia syndrome. These biomarkers have high diagnostic potential for identifying FMS patients, and their study could lead to the development of objective methods for diagnosing FMS. Studying inflammatory and biochemical markers could also lead to new, more targeted treatments for FMS patients in the future.

## Figures and Tables

**Table 1:** Comparison of demographic characteristics, FIQ scores, and serum inflammatory markers (TNF-α, MMP-3, IL-6, and IL-6R) between patients with FMS and controls.

Parameters	Groups	n/Mean ± SD	Min–Max	*p*-Value
Age (years)	FMS	48.44 ± 8.82	27–74	0.5683
	Control	47.46 ± 8.66	25–74	
Gender (Female/Male)	FMS	62/20	—	0.724
	Control	60/22	—	
FIQ	FMS	58.3 ± 13.7	29.4–84.4	<0.0001
	Control	20.1 ± 11.0	1.5–54.8	
TNF-α (pg/mL)	FMS	367.6 ± 297.9	17.7–1274.7	<0.0001
	Control	136.8 ± 116.4	6.0–628.2	
MMP-3 (ng/mL)	FMS	52.7 ± 39.3	8.9–209.4	<0.0001
	Control	15.3 ± 6.7	9.0–36.3	
IL-6 (pg/mL)	FMS	226.3 ± 183.7	9.8–1028.5	<0.0001
	Control	74.6 ± 30.3	4.8–170.0	
IL-6R (pg/mL)	FMS	20.9 ± 14.8	0.6–80.5	<0.0001
	Control	6.7 ± 4.9	0.3–36.3	

Values are expressed as mean ± SD and range (minimum–maximum). The Mann–Whitney U-test was used to compare groups. The results are considered statistically significant at *p* < 0.05. FIQ: Fibromyalgia Impact Questionnaire; TNF-α: tumor necrosis factor-alpha; MMP-3: Matrix Metalloproteinase-3; IL-6: interleukin-6; IL-6R: interleukin-6 receptor.

**Table 2:** Spearman correlation between FIQ score and serum inflammatory markers in FMS group.

Variable	r_s_	95% CI	*p* Value
TNF-α ↔ FIQ	0.540	0.3679 to 0.6757	<0.0001
MMP-3 ↔ FIQ	0.368	0.1669 to 0.5397	0.0004
IL-6 ↔ FIQ	0.551	0.3822 to 0.6847	<0.0001
IL-6R ↔ FIQ	0.497	0.3168 to 0.6430	<0.0001

Spearman’s rank correlation coefficient method was applied to analyze the correlations between the variables. The values obtained for the correlation coefficient are represented as ‘r_s_’ values, along with their respective 95% confidence intervals. The strength of correlation is classified as weak (0.20–0.39), moderate (0.40–0.59), or strong (0.60–0.79). FIQ: Fibromyalgia Impact Questionnaire; TNF-α: tumor necrosis factor-alpha; MMP-3: Matrix Metalloproteinase-3; IL-6: interleukin-6; IL-6R: interleukin-6 receptor.

**Table 3:** Spearman correlation between FIQ score and routine biochemical parameters in FMS group.

Parameter	r_s_	*p*-Value	95% CI
GFR ↔ FIQ	0.155	0.148	−0.06172 to 0.3570
Uric Acid ↔ FIQ	0.467	<0.001	0.2752 to 0.6155
AST ↔ FIQ	0.119	0.266	−0.1406 to 0.2855
ALT ↔ FIQ	0.051	0.637	−0.1653 to 0.2621
CRP ↔ FIQ	0.183	0.085	−0.03202 to 0.3826
Vit. B12 ↔ FIQ	−0.215	0.043	−0.4102 to −0.0007
Vitamin D ↔ FIQ	−0.620	<0.001	−0.7356 to −0.4665
CK ↔ FIQ	0.654	<0.001	0.5121 to 0.7619
fT4 ↔ FIQ	0.024	0.822	−0.1910 to 0.2372
TSH ↔ FIQ	0.047	0.663	−0.1691 to 0.2585

Spearman’s rank correlation coefficient method was applied to analyze the correlations between the variables. The values obtained for the correlation coefficient are represented as ‘r_s_’ values, along with their respective 95% confidence intervals. The strength of correlation is classified as weak (0.20-0.39), moderate (0.40-0.59), or strong (0.60-0.79). FIQ: Fibromyalgia Impact Questionnaire; TNF-α: tumor necrosis factor-alpha; MMP-3: Matrix Metalloproteinase-3; IL-6: interleukin-6; IL-6R: interleukin-6 receptor; GFR: Glomerular Filtration Rate; AST: Aspartate Aminotransferase; ALT: Alanine Aminotransferase; CRP: C-reactive Protein; CK: Creatine Kinase; fT4: Free Thyroxine; TSH: Thyroid-Stimulating Hormone; Vit. B12: Vitamin B12.

**Table 4:** Diagnostic Performance of Serum Biomarkers in Fibromyalgia Syndrome.

Biomarker	AUC	Cut-Off	Sensitivity (%)	Specificity (%)	95% CI	*p*-Value
TNF-α	0.9717	>82.95	95.51	97.75	0.9409–1.0000	<0.0001
MMP-3	0.8283	>41.94	79.78	89.89	0.7566–0.9000	<0.0001
IL-6	0.9530	>82.28	94.38	97.75	0.9103–0.9958	<0.0001
IL-6R	0.9511	>40.07	89.89	91.01	0.9177–0.9846	<0.0001

ROC curve analysis was performed to evaluate the diagnostic performance of the investigated biomarkers. Diagnostic performance was interpreted according to AUC values as follows: excellent (>0.90), good (0.80–0.90), fair (0.70–0.80), and poor (<0.70). AUC: Area Under the Curve; CI: Confidence Interval; IL-6: Interleukin-6; IL-6R: Interleukin 6 Receptor; MMP-3: Matrix Metalloproteinase-3; TNF-α: Tumor Necrosis Factor-alpha.

## Data Availability

The data supporting the findings of this study can be obtained from the corresponding author upon request.

## References

[1] Gupta A., Silman A.J. (2004). Psychological stress and fibromyalgia: A review of the evidence suggesting a neuroendocrine link. Arthritis Res. Ther..

[2] Kocyigit B.F., Akyol A. (2022). Fibromyalgia syndrome: Epidemiology, diagnosis and treatment. Reumatologia.

[3] Siracusa R., Paola R.D., Cuzzocrea S., Impellizzeri D. (2021). Fibromyalgia: Pathogenesis, Mechanisms, Diagnosis and Treatment Options Update. Int. J. Mol. Sci..

[4] Forseth K.O., Gran J.T., Husby G. (1997). A population study of the incidence of fibromyalgia among women aged 26–55 yr. Br. J. Rheumatol..

[5] Güleç H., Sayar K., Güleç M.Y. (2007). Fibromiyaljide Tedavi Arayişinin Psikolojik Etkenlerle Ilişkisi [The relationship between psychological factors and health care-seeking behavior in fibromyalgia patients]. Turk. Psikiyatr. Derg..

[6] Wolfe F., Smythe H.A., Yunus M.B., Bennett R.M., Bombardier C., Goldenberg D.L., Tugwell P., Campbell S.M., Abeles M., Clark P. (1990). The American College of Rheumatology 1990 criteria for the classification of fibromyalgia. Arthritis Rheum..

[7] Wolfe F., Clauw D.J., Fitzcharles M.A., Goldenberg D.L., Häuser W., Katz R.L., Mease P.J., Russell A.S., Russell I.J., Walitt B. (2016). 2016 Revisions to the 2010/2011 fibromyalgia diagnostic criteria. Semin. Arthritis Rheum..

[8] Russell A.S., Gervais R. (2002). Cognitive function in fibromyalgia: Comment on the article by Park et al. Arthritis Rheum..

[9] Dalkılıç E., Gül B., Alkış N. (2012). Interleukin-6: One of the leading actors on inflammation. J. Uludağ Univ. Med. Fac..

[10] Jurado-Priego L.N., Cueto-Ureña C., Ramírez-Expósito M.J., Martínez-Martos J.M. (2024). Fibromyalgia: A review of the pathophysiological mechanisms and multidisciplinary treatment strategies. Biomedicines.

[11] O’Mahony L.F., Srivastava A., Mehta P., Ciurtin C. (2021). Is fibromyalgia associated with a unique cytokine profile? A systematic review and meta-analysis. Rheumatology.

[12] de Gruijter N.M., Jebson B., Rosser E.C. (2022). Cytokine production by human B cells: Role in health and autoimmune disease. Clin. Exp. Immunol..

[13] Taga T., Hibi M., Hirata Y., Yamasaki K., Yasukawa K., Matsuda T., Hirano T., Kishimoto T. (1989). Interleukin-6 triggers the association of its receptor with gp130. Cell.

[14] Heinrich P.C., Behrmann I., Haan S., Hermanns H.M., Müller-Newen G., Schaper F. (2003). Principles of interleukin-6-type cytokine signalling and its regulation. Biochem. J..

[15] Ribbens C., Martin y Porras M., Franchimont N., Kaiser M.J., Jaspar J.M., Damas P., Houssiau F.A., Malaise M.G. (2002). Increased matrix metalloproteinase-3 serum levels in rheumatic diseases: Relationship with synovitis and steroid treatment. Ann. Rheum. Dis..

[16] García-Domínguez M. (2025). Fibromyalgia and Inflammation: Unrevealing the Connection. Cells.

[17] Gyorfi M., Rupp A., Abd-Elsayed A. (2022). Fibromyalgia Pathophysiology. Biomedicines.

[18] Üçeyler N., Häuser W., Sommer C. (2011). Cytokines in fibromyalgia syndrome: A systematic review and meta-analysis. BMC Musculoskelet. Disord..

[19] Häuser W., Ablin J., Fitzcharles M.A., Littlejohn G., Luciano J.V., Usui C., Walitt B. (2015). Fibromyalgia. Nat. Rev. Dis. Prim..

[20] Bazzichi L., Rossi A., Massimetti G., Giannaccini G., Giuliano T., De Feo F., Ciapparelli A., Dell’Osso L., Bombardieri S. (2007). Cytokine patterns in fibromyalgia and their correlation with clinical manifestations. Clin. Exp. Rheumatol..

[21] Andrés-Rodríguez L., Borràs X., Feliu-Soler A., Pérez-Aranda A., Angarita-Osorio N., Moreno-Peral P., Montero-Marin J., García-Campayo J., Carvalho A.F., Maes M. (2020). Peripheral immune aberrations in fibromyalgia: A systematic review and meta-analysis. Brain Behav. Immun..

[22] Sluka K.A., Clauw D.J. (2016). Neurobiology of fibromyalgia and chronic widespread pain. Neuroscience.

[23] Tanaka T., Narazaki M., Kishimoto T. (2014). IL-6 in inflammation, immunity, and disease. Cold Spring Harb. Perspect. Biol..

[24] Rose-John S. (2012). IL-6 trans-signaling via the soluble IL-6 receptor: Importance for the pro-inflammatory activities of IL-6. Int. J. Biol. Sci..

[25] Clauw D.J. (2014). Fibromyalgia: A clinical review. JAMA.

[26] Wang H., Zhou H., Cai Y., Ye Z. (2025). The causal relationship between matrix metalloproteinase-3 and fibromyalgia: A two-sample Mendelian randomization analysis. Medicine.

[27] Meester I., Rivera-Silva G.F., González-Salazar F. (2020). Immune System Sex Differences May Bridge the Gap Between Sex and Gender in Fibromyalgia. Front. Neurosci..

[28] Makrani A.H., Afshari M., Ghajar M., Forooghi Z., Moosazadeh M. (2017). Vitamin D and fibromyalgia: A meta-analysis. Korean J. Pain.

[29] Ismail O., Albdour K., Albdour Z., Jaber K. (2025). Differences in ferritin, vitamin D, and vitamin B12 between fibromyalgia patients and healthy individuals: A systematic review and meta-analysis. Musculoskelet. Care.

[30] Cure O., Kizilkaya B., Ciftel S., Klisic A., Ciftel E., Mercantepe F. (2024). Effect of fibromyalgia on demographic, biochemical, metabolic and inflammatory profiles: A single-centre retrospective study. Clin. Exp. Rheumatol..

[31] Uçeyler N., Valenza R., Stock M., Schedel R., Sprotte G., Sommer C. (2006). Reduced levels of antiinflammatory cytokines in patients with chronic widespread pain. Arthritis Rheum..

[32] Kumbhare D., Hassan S., Diep D., Duarte F.C.K., Hung J., Damodara S., West D.W.D., Selvaganapathy P.R. (2022). Potential role of blood biomarkers in patients with fibromyalgia: A systematic review with meta-analysis. Pain.

[33] Mueller C., Fang Y.D., Jones C., McConathy J.E., Raman F., Lapi S.E., Younger J.W. (2023). Evidence of neuroinflammation in fibromyalgia syndrome: A [18 F]DPA-714 positron emission tomography study. Pain.

